# Human Physiology

**Published:** 1856-10

**Authors:** 


*Human Physiology.* By ROBLEY DUNGLISON, M.D.,L.L.D. &c., &c., &c., with five hundred and thirty-two Illustrations. Eighth Edition, Revised, Modified, and Enlarged, in two Volumes. Philadelphia, Blanchard & Lea, 1856.

We cannot attempt a review or analysis of this work. It constitutes a kind of Encyclopedia of Physiology, in which the doctrines and opinions of the present age, are mingled with those of the past. Dr. DUNGLISON, as our readers are aware, is distinguished for his great learning, rather than for originality of investigation. It is for this reason that his compilations are more valuable than his original works. We say this without wishing to detract in the least from the reputation of this distinguished author, whose name has become so intimately associated with the science of Medicine in this country.

The work is for sale by KEEN & LEE.

